# Toxoplasmosis or left ventricular hypertrabeculation / non-compaction

**Published:** 2012-09-25

**Authors:** J Finsterer, C Stöllberger

**Affiliations:** * Krankenanstalt Rudolfstiftung, Vienna, Austria; ** 2nd Medical Department, Krankenanstalt Rudolfstiftung, Vienna, Austria

## Letter to the Editor

We read with interest the case report by Dobranici et al. about a 23-year-old female with dilated cardiomyopathy and left ventricular hypertrabeculation / non-compaction (LVHT) who also suffered from chronic toxoplasmosis [**1**]. The article raises a number of concerns.

Since LVHT develops during life in single cases (acquired LVHT) and since there is uncertainty about the pathogenesis of LVHT, it cannot be postulated that LVHT is exclusively an embryonic abnormality [**2**].

Due to the association of LVHT with numerous different genetic causes, it is questionable to classify LVHT as cardiomyopathy. More likely, LVHT represents an adaptation mechanism of myocardial involvement in a global genetic defect.

As mentioned by the authors, LVHT may be associated with neuromuscular disorders (NMDs) [**3**]. Did a neurologist for NMD investigate the presented patient? Did the patient suffer from a multi-system neurological disease? Was the family history positive for NMD? 

Since LVHT is frequently associated with chromosomal abnormalities, it would be interesting to know if the phenotype was suggestive of a chromosomal disease or if the patient or any of her relatives was investigated for chromosomal abnormalities. 

Since LVHT occurs familial [**4**], it would be interesting to know if other family members were investigated for ECG abnormalities and LVHT and if LVHT was found in any of them?

Toxoplasma myocarditis in immunocompetent patients is not unknown as demonstrated by several case reports in the literature [**5**]. Possibly, toxoplasmic cysts may mimic LVHT. We have also shown that abscesses due to candida may pretend LVHT on echocardiography [**6**] and reversible LVHT has been reported in a young male with Coxsackie myocarditis [**7**].

Based on which diagnostic procedures, was toxoplasmosis diagnosed in the presented patient? Were any signs of inflammation or myocardial oedema found on cardiac magnetic resonance imaging? Which was the HIV-status of the patient? Toxoplasma myocarditis particularly occurs in HIV-positive patients [**8**]. Which antibiotic treatment did she receive and why for such a long time, which is unusual? Which were the results of the follow-up investigations for serum IgG antibodies against toxoplasma?

For how long was the patient already receiving antibiotic drugs for toxoplasmosis when dilated cardiomyopathy was diagnosed? Assuming that antibiotic treatment for toxoplasmosis was effective, dilated cardiomyopathy should have already been resolved and dilated cardiomyopathy could be attributed to another, most likely, genetic cause. 

Why was the patient anticoagulated? Was atrial fibrillation ever recorded? Did heart failure not resolve under treatment? LVHT in the absence of severe systolic function or atrial fibrillation per se is no indication for oral anticoagulation. Why did the patient receive an ICD? Were severe ventricular arrhythmias ever recorded by long-term ECG recordings?

The follow up period of the patient is unclear in the presentation. Was LVHT also present after the resolution of systolic dysfunction and heart failure? Did ECG abnormalities resolve during follow-up? Which was the long-term outcome of the patient?

Overall, it would be eligible to increase the information about comorbidity in the presented patient and his relatives. Treatment of LVHT should rely on the therapy of comorbidities, such as systolic dysfunction, ventricular arrhythmias, or stroke/embolism. LVHT per se is no indication for cardiac therapy.
